# A Contemporary Review on Heart Failure with Preserved Ejection Fraction: Epidemiology, Diagnosis, and Management

**DOI:** 10.2174/011573403X318646240909072055

**Published:** 2024-09-19

**Authors:** Mahek Shahid, Ramzi Ibrahim, Abdulbaril Olagunju, Martina Mookadam, Farouk Mookadam

**Affiliations:** 1 Department of Medicine, University of Arizona Tucson, Tucson, Arizona, United States;; 2 Sarver Heart Center, University of Arizona Tucson, Tucson, Arizona, United States;; 3 Department of Family Medicine, Mayo Clinic, Phoenix, Arizona, United States;; 4 Department of Cardiology, Banner University Medical Center, Phoenix, Arizona, United States

**Keywords:** Heart failure, diastolic, congestion, cardiovascular, algorithmic frameworks, metabolic syndrome

## Abstract

Heart failure with preserved ejection fraction (HFpEF) includes almost half of heart failure cases typified by a specific clinical syndrome. Despite diagnostic and management advances, HFpEF still presents a diagnostic challenge and a paucity of therapies specifically aimed at enhancing survival and improving quality of life is still lacking. This review elucidates the diagnostic complexity of HFpEF, highlighting the use of both subjective and objective criteria within algorithmic frameworks. It also examines the significant impact of comorbidities on the progression of HFpEF. Additionally, we explore the latest evidence on targeting these comorbidities therapeutically, although the benefits to mortality are still limited.

## 1 INTRODUCTION

Heart failure with preserved ejection fraction (HFpEF) is highly prevalent, including up to fifty percent of heart failure diagnoses [1]. Although it shares similarities with heart failure with reduced ejection fraction (HFrEF), HFpEF tends to present with more subtle symptoms. Nonetheless, the outcomes are just as severe, with comparable rates of mortality and hospitalization [2]. HFpEF patients also often endure a higher burden of comorbidities, adding to the complexity of management [3]. The diagnosis and treatment of HFpEF are challenging, as HFpEF functions more as a clinical syndrome than a condition stemming from a single cause. This view is supported by the absence of disease-specific treatments emerging from multiple randomized controlled trials that significantly improve prognosis. In this review, we synthesize the current evidence on diagnostic criteria, epidemiology and risk factors, and therapeutic considerations with an emphasis on recent clinical trials concerning HFpEF.

## 2 EPIDEMIOLOGY AND RISK FACTORS

Fifty percent of HF in the US is HFpEF [1]. Trends have shown an increasing prevalence of about one percent annually [4, 5]. Risk of developing HFpEF is similar among males and females; however, overall prevalence is higher in females [5, 6]. This increase is due to pre-disposing conditions for HFpEF, such as hypertension, DM, obesity, metabolic syndrome, and coronary artery disease (CAD) [7].

### 2.1 Systemic Hypertension

Systemic hypertension increases left ventricular afterload, subsequently resulting in pressure-related left ventricular remodeling expressed by increased microtubule density and changes in the extracellular matrix [8-10]. Progressive hypertrophy and fibrosis via abnormal calcium handling, hyperphosphorylation of titin protein, increased collagen turnover, and microtubule disarray result in impaired relaxation and increased myocardial stiffness, leading to diastolic dysfunction [11, 12]. Once left ventricular hypertrophy (LVH) ensues, the risk of developing heart failure increases. For example, every one percent increase in left ventricular mass above the normal limits has been associated with a one percent increase in the incidence of heart failure [13].

### 2.2 Metabolic Syndrome

Metabolic syndrome increases the risk of HFpEF [14]. Inflammation, a characteristic feature of metabolic syndrome, results in elevations in inflammatory cytokines which are more pronounced in HFpEF compared to HFrEF [15]. The pro-inflammatory cytokines, TNF-alpha, IL-6, and adiponectin, increase endothelial production of reactive oxygen species (ROS), decrease nitric oxide availability, and increase peroxynitrite. This leads to increased cardiac remodeling and hypertrophy through decreased cyclic guanosine monophosphate (cGMP) levels and protein kinase G (PKG) activity. Through hypertrophy and fibrosis stimulated by the inflammatory state, the myocardial tissue stiffens, resulting in impaired diastolic relaxation and the development of HFpEF.

### 2.3 Renal Dysfunction

Chronic kidney disease (CKD) can predispose to HFpEF through arterial stiffening, volume expansion, renin-angiotensin-aldosterone system (RAAS) activation, and hypertension, all of which increase afterload [16]. CKD is a pro-inflammatory state that can lead to cardiac remodeling, including myocardial hypertrophy and interstitial fibrosis [17]. Beyond direct inflammatory markers, impaired renal clearance causes retention of uremic toxins, which increase ROS in vascular endothelium, promoting oxidative stress [18]. Fibroblast growth factor-23 (FGF23) production increases in renal dysfunction to compensate for disturbances in calcium-phosphate metabolism. While FGF23 signaling receptor activation in the kidneys and parathyroid gland allows for alteration in gene expression involving the vitamin D and phosphate balance, receptor activation in cardiomyocytes induces pro-hypertrophic signaling pathways promoting LVH [19]. Renal dysfunction patients show greater LVH, RVH, LV mass, and overall LV dysfunction [4, 20].

### 2.4 Coronary Artery Disease

Coronary artery disease (CAD) is prevalent in approximately 40-50% of patients with HFpEF [21]. Patients with HFpEF and CAD had a lower EF and higher mortality compared to patients with HFpEF without CAD [22]. The intervention in CAD in these populations has shown mixed results [22, 23]. Further studies are warranted regarding revascularization in patients with HFpEF.

HFpEF has also been linked to coronary microvascular dysfunction. Impaired coronary flow reserve, despite the absence of obstructive CAD is present in up to 75% of patients with HFpEF [24]. Endothelial dysfunction leads to decreased nitric oxide availability, which promotes proliferation of fibroblasts and myofibroblasts, as well as hyperphosphorylation of the titin, impairing cardiomyocyte relaxation [25, 26]. Biopsies from patients with HFpEF show inflamed microvascular endothelium that may cause micro-infarcts, resulting in cardiac interstitial fibrosis [25].

### 2.5 Advanced Age

With the aging population and advancements in heart failure therapies, the prevalence of HF continues to rise [27]. This increase particularly impacts elderly populations who have known risk factors for HF and a greater burden of comorbidities [28, 29]. Despite the significant impact of HF on elderly populations, these groups are poorly represented in clinical trials [30, 31]. Furthermore, the use of guideline-directed medical therapies is often underutilized in the elderly [28]. This gap between contemporary evidence in HF treatments and the representation of elderly data in clinical practice likely exacerbates the burden of HF and leads to preventable poor outcomes [29]. For instance, a study that evaluated hemodynamic and echocardiographic categories, which have been poorly characterized in elderly populations with HF, identified several factors associated with adverse prognostic indications. These factors included the “cold-dry” phenotype, male sex, low eGFR, and higher sodium levels [32].

## 3 DIAGNOSTIC CRITERIA

Diagnosis of HFpEF requires the combination of signs and symptoms of volume overload, an EF ≥50% with structural and/or functional abnormalities on transthoracic echocardiogram at rest or exercise, and the presence of elevated natriuretic peptides [33]. Dyspnea with or without edema is the most common presenting symptom in patients with HFpEF [33]. However, other cardiac and non-cardiac etiologies of dyspnea and edema besides HFpEF should be considered. Natriuretic peptide levels and parameters of elevated left ventricular filling pressures obtained via TTE or invasively by a right heart catheterization can be unequivocal in a portion of patients with HFpEF [33]. Therefore, point-based algorithms that incorporate signs and symptoms, risk factors, and objective evidence of elevated left ventricular filling pressures have been proposed to better identify dyspneic patients with HFpEF [33]. Two recently validated algorithms are the H2FPEF and the HFA-PEFF, proposed by the European Society of Cardiology’s Heart Failure Association [33].

The H2FPEF algorithm has 6 components, which are described in Table **1**. A H2FPEF score of ≥ 6 or more is indicative of HFpEF. The H2FPEF algorithm is user-friendly at the point of care and has greater sensitivity compared to the HFA-PEFF algorithm [34]. Despite including more echocardiographic measurements of diastology in the HFA-PEFF algorithm, the HFA-PEFF was associated with a higher rate of false negatives compared to the H2FPEF score [34]. The HFA-PEFF is a better predictor of mortality in patients with cardiac amyloidosis compared to the H2FPEF algorithm [35]. The HFA-PEFF algorithm uses a stepwise approach [36]. Step 1 is the pretest assessment including signs and symptoms of HF, comorbidities and risk factors, and electrocardiographic clues such as left ventricular hypertrophy, left ventricular (LV) diameter and left ventricular ejection fraction (LVEF), natriuretic peptides levels, and exercise tests [36]. If HFpEF is suspected, then step 2 is pursued, which includes a comprehensive evaluation of TTE parameters and natriuretic peptide levels [36]. The TTE parameters include the conventional ASE and ESC recommendations displayed in Table **2** [36]. A score ≥5 is diagnostic of HFpEF, whilst a score < 5 requires step 3, including stress echocardiography or invasive hemodynamic measurements *via* right and left heart catheterization may be considered. An E/e’ ratio ≥15 adds 2 points to the overall score calculated in step 2 and an E/e’ ratio ≥15 with a peak TR velocity >3.4m/s adds 3 points to the overall score [36]. Furthermore, pulmonary capillary wedge pressure ≥15mmHg at rest or ≥25mmHg after stress is diagnostic of HFpEF [36]. After diagnosis, step 4 is taken to identify the possible etiology to allow for targeted therapy [36].

## 4 TREATMENT OF HFPEF

**Fig. (1):**
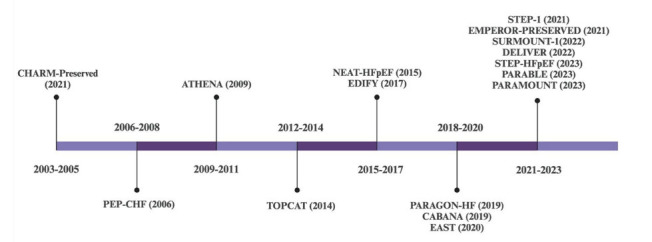
Timeline representation of clinical trials. Figure includes the timeline representation of the relevant major clinical trials since 2003 that have impacted the therapeutic approach to HFpEF management. **Abbreviations:** HFpEF=heart failure with preserved ejection fraction. Source: Created with Biorender.com.

Treatment objectives for HFpEF patients include symptom reduction, mortality decrease, enhancement of quality of life and functional capacity, and minimizing hospitalization risks. The intensity of heart failure and related comorbidities often guide the need for clinical assessments. Regular evaluations, coupled with structured exercise regimens, cardiac rehabilitation, and nutritional guidance, are instrumental in meeting the multifaceted treatment goals for those affected by HFpEF. The therapeutic approach for HFpEF is the management of symptoms acutely, with low dose diuretics to allow decongestion, excluding CAD, and revascularizing when necessary. Then, turning attention to the comorbidities, including obesity, hypertension, DM, atrial fibrillation, CAD, and CKD. Several trials highlight beneficial medications for use in HFpEF (Fig. **1** and Table **3**).

### 4.1 Obesity

Recent pharmacological developments have offered promising results for the management of HFpEF. More than half of all individuals with HFpEF are overweight [37]. Weight reduction has been associated with reduced heart rate, blood pressure, mean pulmonary artery pressure, and pulmonary capillary wedge pressures [38]. Incorporating resistance and aerobic exercise training along with caloric restriction not only enhances the quality of life and peak oxygen consumption during exercise for those with HFpEF but also helps in preserving skeletal muscle, reinforcing the importance of weight management in HFpEF treatment [39].

Recently, GLP-1 agonists, previously recognized for their cardiovascular benefits in diabetic populations, have gained attention for their role in weight management. The STEP-1 trial showed significant weight loss with semaglutide in non-diabetic individuals with obesity [40]. This trial randomized 1,961 patients with a BMI > 30 without diabetes to receive either semaglutide or placebo. The mean change in body weight at week 68 was significantly greater in those who received semaglutide (-14.9%) versus placebo (-2.4%). This was further supported by the SURMOUNT-1 trial, which showed weight loss with tirzepatide [41]. This trial randomized 2,539 patients with a body mass index ≥30 or a BMI ≥ 27 with one weight-related complication to receive tirzepatide or a placebo. By week 72, the mean percentage change in weight was -20.9% in the tirzepatide arm and -3.1% with the placebo (*p*<0.001). Yet, the use of these medications in the HFpEF population has been met with caution due to the limited data on their effects in this specific group and concerns over potential muscle mass loss, which could exacerbate deconditioning.

The STEP-HFpEF trial in 2023 addressed this gap by specifically assessing the efficacy of semaglutide in patients with HFpEF and obesity [42]. This trial randomized 529 patients with HFpEF and a body mass index ≥30 to receive semaglutide or placebo for 52 weeks. Results showed that semaglutide significantly improved functional capacity and symptoms compared to placebo. Despite these positive outcomes, the debate continues due to the potential for muscle mass loss, underlining the need for a nuanced approach to using these agents in the HFpEF population.

### 4.2 Hypertension

Hypertension remains the most modifiable risk factor [43]. Following the ACC/AHA 2017 guidelines, the therapeutic target for these patients is a systolic blood pressure˂130 mmHg, with evidence derived from studies showing that intensive blood pressure management reduces cardiovascular events [44, 45].

It is common for individuals with concurrent hypertension and HFpEF to necessitate multiple antihypertensive medications. Beta blockers, may impair functional capacity if the heart rate is lowered excessively, impairing cardiac output. This was shown in the myPACE trial, demonstrating that patients with HFpEF benefit from personalized accelerated pacing, which significantly improved quality of life and physical activity levels compared to usual care [46].

Angiotensin-converting-enzyme (ACE) inhibitors, angiotensin II receptor blockers (ARBs), angiotensin receptor/neprilysin inhibitors (ARNIs), thiazides, and mineralocorticoid receptor antagonists (MRAs) are among the recommended medications. The CHARM-Preserved trial was among the first trials to explore the benefits of an ARB in HFpEF [47]. This trial randomized 3,023 patients to receive either candesartan or placebo and found that heart failure hospitalizations were lower in patients treated with candesartan (n=230) versus with placebo (n=279) (p=0.017). Following this, PEP-CHF trial randomized 850 patients with HFpEF to receive perindopril or a placebo [48]. Despite the PEP-CHF study being underpowered, it suggested that perindopril may improve symptoms and exercise capacity. Subsequently, ARNI use was evaluated for the HFpEF population in the PARAGON-HF trial, which randomized 4,822 patients with HFpEF to receive either sacubitril–valsartan or valsartan alone [49]. This trial found that while sacubitril-valsartan did not reduce mortality or heart failure hospitalizations, it improved NYHA class and preserved renal function. Additionally, a post-hoc analysis of the PARAGON trial indicated that sacubitril-valsartan could be beneficial for resistant hypertension [50]. The subsequent PARABLE and PARAMOUNT trials further supported the efficacy of sacubitril-valsartan in improving left atrial volume index and global circumferential strain in patients with HFpEF [51, 52].

The TOPCAT trial revealed no significant difference in major outcomes in HFpEF [53]. However, a post-hoc analysis suggested that geographical differences in patient populations could explain the varied results, as participants from the Americas showed a significant benefit in the spironolactone group [54]. This discrepancy was likely related to patient heterogeneity as patients from Georgia and Russa were younger and had lower rates of diabetes and atrial fibrillation, lower left ventricular ejection fractions, and higher diastolic blood pressures.

### 4.3 Diabetes Mellitus

Diabetes mellitus is common in patients with HFpEF, and its presence exacerbates the risk of heart failure hospitalizations and mortality. As such, diabetes management is a crucial aspect of comprehensive HFpEF care. The American Diabetes Association suggests that individualized hemoglobin A1c targets ranging between 7.5-8.5% should be considered, taking into account factors such as comorbidity burden, cognitive function, polypharmacy, and the patient prognosis [55].

SGLT-2 inhibitors have emerged as a first-line treatment for diabetes in the absence of contraindications, particularly due to their pronounced cardiovascular benefits in the HFpEF population [56]. EMPEROR-Preserved randomized 5,988 patients with HFpEF to receive empagliflozin or placebo [57]. This trial demonstrated a significant reduction in the composite endpoint of cardiovascular death or heart failure hospitalization with empagliflozin, driven by a decrease in heart failure hospitalizations [58]. Similarly, the DELIVER trial confirmed the efficacy of SGLT-2 inhibitors by randomizing 6,263 patients with HFpEF to receive dapagliflozin or placebo [59]. Results revealed that dapagliflozin significantly reduced cardiovascular death and HF events in patients with HFpEF.

GLP-1 agonists are recommended, especially for HFpEF patients with concurrent atherosclerotic coronary disease [60]. Conversely, certain dipeptidyl peptidase-4 inhibitors like saxagliptin and alogliptin, as well as thiazolidinediones, are linked to adverse effects such as fluid retention, weight gain, and an increased rate of heart failure hospitalizations [61, 62]. More information regarding the benefits of GLP-1 agonists is described in the *Obesity* section under Treatment.

### 4.4 Atrial Fibrillation

Atrial fibrillation is associated with a higher risk of mortality and hospitalization in individuals with HFpEF [63]. Evidence from a post-hoc analysis of the DELIVER trial indicated an increased incidence of the primary composite endpoint—cardiovascular death or heart failure hospitalization—in patients with AF and HFpEF. Notably, the cardiovascular benefits of dapagliflozin in reducing this composite endpoint were consistent, irrespective of the presence of AF [64].

Recent trends favor rhythm control strategies, particularly in patients with newly diagnosed AF, over rate control strategies. The ATHENA trial in 2009 showed that dronedarone decreased cardiovascular events in patients with atrial fibrillation across a range of ejection fractions, including those with comorbid HFpEF [65]. The CABANA trial in 2019 randomized 2,204 patients with symptomatic atrial fibrillation to receive either catheter ablation or conventional medical therapy [66]. While the CABANA trial was inconclusive regarding overall survival in an intention-to-treat analysis, further analyses suggested a significant survival benefit in the heart failure subgroup, notably those with preserved ejection fraction [66, 67]. This was followed by the EAST trial in 2020, which randomized patients with atrial fibrillation and, many of whom had HFpEF, to receive early rhythm control or usual care [68, 69]. This trial demonstrated advantages of early rhythm control using pharmacotherapy over usual care in reducing cardiovascular events. A smaller Australian trial of 31 patients was randomized to receive either catheter ablation or medical therapy for AF in patients with HFpEF, which showed that catheter ablation improved quality of life and exercise hemodynamics [70].

Nondihydropyridine calcium channel blockers and beta-blockers remain the cornerstone of rate control therapy in patients with AF and HFpEF. Care is warranted when administering these agents as aggressive rate control may lead to a decline in functional capacity, compounded by symptoms such as lethargy, hypotension, and dizziness [71].

### 4.5 Coronary Artery Disease

The approach to acute coronary syndromes in individuals with HFpEF aligns with the standard revascularization guidelines [72]. However, there is a notable absence of prospective trial data on the effects of revascularization in the HFpEF population. For chronic CAD disease and associated angina, clinicians may prescribe antianginal agents cautiously. Beta-blockers have been shown to augment chronotropic incompetence and decrease functional capacity, which was supported by the findings of the PRESERVE-HR trial. This trial randomized 52 patients with HFpEF and known treatment with beta-blockers to either discontinuation of the beta-blocker or continuation of it. Primary outcomes included peak oxygen consumption, which increased significantly among the beta-blocker discontinuation group. However, not all patients in this trial were on beta-blockade as an anti-anginal. Furthermore, the NEAT-HFpEF trial randomized 110 patients to receive isosorbide mononitrate or placebo and did not show a significant improvement in the primary endpoint of daily activity level in the treatment group [73]. Consequently, current AHA/ACC/HFSA guidelines advise against using nitrates for enhancing exercise capacity in HFpEF [74]. Similarly, ivabradine was not shown to be beneficial in HFpEF, as evidenced by EDIFY [75]. This trial randomized 179 patients with HFpEF to ivabradine or placebo. There were no significant differences in NT-proBNP levels, E/e' ratio, or 6-minute walk test distances between the 2 groups after 8 months.

### 4.6 Chronic Kidney Disease

Management of chronic kidney disease in patients with HFpEF involves a multidisciplinary approach, with guidance from both cardiology and nephrology. The therapeutic armamentarium includes ACE inhibitors, ARBs, SGLT2 inhibitors, and MRAs, which aim to mitigate the progression of renal impairment. The PARAGON-HF trial in 2019 revealed the superior renal protective effects of ARNI over ARB in patients with HFpEF [49]. This protective effect on renal function was further corroborated by the EMPEROR-Preserved trial, which reported a significantly reduced rate of eGFR decline in the empagliflozin group compared to placebo [57].

## CONCLUSION

HFpEF is a complex diagnosis, and its etiology is driven by many proinflammatory cytokines, influencing its development and progression. A novel approach to prevention and treatment involves a comprehensive evaluation of associated conditions such as hypertension, diabetes, kidney dysfunction, and coronary artery disease. Despite the variety of therapeutic agents available that can improve functional capacity and reduce heart failure hospitalizations, there is only limited benefit on survival. Prevention of HFpEF then seems to be the key focus, which can be accomplished through national efforts at reducing the comorbidity burden. The role of anti-inflammatory medications has yet to be formally tested in clinical trials. Ongoing research is essential to deepen our understanding of underlying mechanisms in HFpEF and to influence future therapeutic targets that may improve survival outcomes and quality of life for patients with HFpEF.

## Figures and Tables

**Table 1:** H_2_FPEF Algorithm. Table includes the scoring system for the H_2_FPEF algorithm.

**H_2_FPEF Algorithm**	
Clinical variables	Points
Heavy (BMI) >30kg/m2	2
Hypertension (≥2 drugs)	1
Atrial Fibrillation	3
Pulmonary hypertension (PASP >35mmHg)	1
Age >60 years	1
E/e' ratio	1
H2FPEF score ≥6: HFpEF	

**Abbreviations:** BMI=body mass index, PASP=pulmonary artery systolic pressure.

**Table 2:** HFA-PEFF Algorithm. Table includes the scoring system including echocardiographic parameters of function and morphology and natriuretic peptide levels included in the HFA-PEFF algorithm.

**HFA-PEFF Algorithm (Step 2)**	
Major criteria (2 points)	Minor criteria (1 point)
**Echocardiographic parameters of function**	
Septal e' <7cm/s for age <75years	Average E/e' 9-14
Lateral e' <10cm/s for age <75years	GLS <16%
Septal e' <5cm/s for age ≥ 75years	
Lateral e' <7cm/s for age ≥ 75years	
Average E/e' ≥15	
TR velocity >2.8m/s	
PASP >35mmHg	
**Echocardiographic parameters of morphology**	
LAVI >34mL/m2 in SR	LAVI 29-34mL/m2 in SR
LAVI >40mL/m2 in AF	LAVI 34-40mL/m2 in AF
LVMI ≥149g/m2 in M & RWT >0.42	LVMI ≥115g/m2 in M
LVMI ≥122g/m2 in W & RWT >0.42	LVMI ≥95g/m2 in W
	RWT >0.42
**Natriuretic peptides levels in SR**	
NT-proBNP >220 pg/mL	NT-proBNP 125-220 pg/mL
BNP >80 pg/mL	BNP 35-80 pg/mL
**Natriuretic peptides levels in AF**	
NT-proBNP >600 pg/mL	NT-proBNP 365-660 pg/mL
BNP >240 pg/mL	BNP 105-240 pg/mL
≥5 points: HFpEF; 2-4 points: diastolic stress test or assess invasive hemodynamics	

**Abbreviations:** AF=atrial fibrillation, GLS=Global Longitudinal Strain, LVMI=Left Ventricular Mass Index, M=Men, W=Women, RWT=Relative Wall Thickness, SR=Sinus Rhythm.

**Table 3:** Clinical Trials Related to HFpEF. Table depicts many of the relevant major clinical trials since 2003 that have impacted the therapeutic approach to HFpEF management.

**Clinical Trial**	**Year Published**	**Number of Patients**	**Population**	**Intervention**	**Major Findings**
CHARM-Preserved	2003	3023	Population with HFpEF	Candesartan *vs* placebo	Reduction in heart failure hospitalizations with Candesartan
PEP-CHF	2006	850	Population with HFpEF	Perindopril *vs* placebo	Improvement in symptoms and exercise capacity with perindopril
ATHENA	2009	4628	Population with AF, including comorbid HFpEF	Dronedarone *vs* placebo	Reduction in cardiovascular hospitalization or death with dronedarone
TOPCAT	2014	3445	Population with HFpEF	Spironolactone *vs* placebo	No difference in cardiac arrest, heart failure hospitalization, death from cardiovascular causes
NEAT-HFpEF	2015	110	Population with HFpEF	Isosorbide mononitrate *vs* placebo	No difference in quality of life or exercise capacity with isosorbide mononitrate
EDIFY	2017	179	Patients with HFpEF	Ivabradine *vs* placebo	No difference in NT-proBNP concentration, echo-Doppler E/e' ratio, or distance on 6-min walking test with ivabradine
PARAGON-HF	2019	4822	Patients with HFpEF	Sacubitril-valsartan *vs* valsartan	No difference in HF hospitalization or cardiovascular death but improvement in NYHA class and preserved renal function with sacubitril-valsartan
CABANA	2019	2204	Population with AF, including comorbid HFpEF	Catheter ablation *vs* usual medical therapy	No difference in death, stroke, serious bleeding, or cardiac arrest with catheter ablation
EAST	2020	2789	Population with AF, including comorbid HFpEF	Early rhythm control *vs* usual care	Reduction in cardiovascular events with early rhythm control
STEP-1	2021	1961	Non-diabetic individuals with obesity	Semaglutide *vs* placebo	Sustained reduction in body weight with semaglutide
EMPEROR-Preserved	2021	5988	Population with HFpEF	Empagliflozin *vs* placebo	Decreased cardiovascular death or heart failure hospitalization with empagliflozin
PRESERVE-HR	2021	52	Population with HFpEF on beta-blockers	Discontinuation of beta-blockers *vs* continuation of beta-blockers	Discontinuation of beta-blockers showed an increase in peak oxygen consumption
SURMOUNT-1	2022	2539	Non-diabetic individuals with obesity	Tirzepatide *vs* placebo	Sustained reduction in body weight with tirzepatide
DELIVER	2022	6263	Population with HFpEF	Dapagliflozin *vs* placebo	Decreased cardiovascular death or heart failure hospitalization with dapagliflozin
STEP-HFpEF	2023	529	HFpEF and obesity	Semaglutide *vs* placebo	Improvement in physical functioning, decreased symptoms, and greater weight loss with semaglutide
PARABLE	2023	250	Population with HFpEF	Sacubitril/valsartan *vs* valsartan	Increase in left atrial volume index
PARAMOUNT	2023	301	Population with HFpEF	Sacubitril/valsartan *vs* valsartan	Improvement in global circumferential strain

**Abbreviations:** HFpEF=heart failure with preserved ejection fraction.

## LIST OF ABBREVIATIONS

HFpEF: Heart failure with Preserved Ejection Fraction
LVH: Left Ventricular Hypertrophy
ROS: Reactive Oxygen Species
PKG: Protein Kinase G
cGMP: Cyclic Guanosine Monophosphate
CAD: Coronary Artery Disease
CKD: Chronic Kidney Disease
RAAS: Renin-Angiotensin-Aldosterone System
FGF23: Fibroblast Growth Factor-23
ARNIs: Angiotensin Receptor/Neprilysin Inhibitors
ACE: Angiotensin-Converting-Enzyme
ARBs: Inhibitors, Angiotensin II Receptor Blockers
